# Temporal Irreversibility of Large-Scale Brain Dynamics in Alzheimer’s Disease

**DOI:** 10.1523/JNEUROSCI.1312-22.2022

**Published:** 2023-03-01

**Authors:** Josephine Cruzat, Ruben Herzog, Pavel Prado, Yonatan Sanz-Perl, Raul Gonzalez-Gomez, Sebastian Moguilner, Morten L. Kringelbach, Gustavo Deco, Enzo Tagliazucchi, Agustín Ibañez

**Affiliations:** ^1^Latin American Brain Health Institute (BrainLat), Universidad Adolfo Ibañez, 7911328, Santiago, Chile; ^2^Fundación para el Estudio de la Conciencia Humana (ECoH), 7550000, Santiago, Chile; ^3^Department of Physics, University of Buenos Aires, C1428EGA, Buenos Aires, Argentina; ^4^National Scientific and Technical Research Council (CONICET), C1033AAJ, Buenos Aires, Argentina; ^5^Cognitive Neuroscience Center (CNC), Universidad de San Andrés, C116ABJ, Buenos Aires, Argentina; ^6^Center for Brain and Cognition, Computational Neuroscience Group, Universitat Pompeu Fabra, 08005 Barcelona, Spain; ^7^Global Brain Health Institute, University of California, San Francisco, San Francisco, California 94143; ^8^Global Brain Health Institute, Trinity College, Dublin 2, Ireland; ^9^Department of Psychiatry, University of Oxford, Oxford OX3 7JX, United Kingdom; ^10^Center for Music in the Brain, Department of Clinical Medicine, Aarhus University, 8000 Århus, Denmark; ^11^Centre for Eudaimonia and Human Flourishing, Linacre College, University of Oxford, Oxford OX3 9BX, United Kingdom; ^12^Department of Information and Communication Technologies, Universitat Pompeu Fabra, 08018 Barcelona, Spain; ^13^Institució Catalana de la Recerca i Estudis Avancats (ICREA), 08010 Barcelona, Spain; ^14^Department of Neuropsychology, Max Planck Institute for Human Cognitive and Brain Sciences, D-04303 Leipzig, Germany; ^15^School of Psychological Sciences, Monash University, Melbourne 3168, Australia; ^16^Trinity College Institute of Neuroscience, Trinity College Dublin, Dublin 2, Ireland; ^17^Escuela de Fonoaudiología, Facultad de Odontología y Ciencias de la Rehabilitación, Universidad San Sebastián, Santiago, Chile

**Keywords:** Alzheimer’s disease, dynamic networks, EEG, fMRI, irreversibility dynamics, machine learning

## Abstract

Healthy brain dynamics can be understood as the emergence of a complex system far from thermodynamic equilibrium. Brain dynamics are temporally irreversible and thus establish a preferred direction in time (i.e., arrow of time). However, little is known about how the time-reversal symmetry of spontaneous brain activity is affected by Alzheimer's disease (AD). We hypothesized that the level of irreversibility would be compromised in AD, signaling a fundamental shift in the collective properties of brain activity toward equilibrium dynamics. We investigated the irreversibility from resting-state fMRI and EEG data in male and female human patients with AD and elderly healthy control subjects (HCs). We quantified the level of irreversibility and, thus, proximity to nonequilibrium dynamics by comparing forward and backward time series through time-shifted correlations. AD was associated with a breakdown of temporal irreversibility at the global, local, and network levels, and at multiple oscillatory frequency bands. At the local level, temporoparietal and frontal regions were affected by AD. The limbic, frontoparietal, default mode, and salience networks were the most compromised at the network level. The temporal reversibility was associated with cognitive decline in AD and gray matter volume in HCs. The irreversibility of brain dynamics provided higher accuracy and more distinctive information than classical neurocognitive measures when differentiating AD from control subjects. Findings were validated using an out-of-sample cohort. Present results offer new evidence regarding pathophysiological links between the entropy generation rate of brain dynamics and the clinical presentation of AD, opening new avenues for dementia characterization at different levels.

**SIGNIFICANCE STATEMENT** By assessing the irreversibility of large-scale dynamics across multiple brain signals, we provide a precise signature capable of distinguishing Alzheimer’s disease (AD) at the global, local, and network levels and different oscillatory regimes. Irreversibility of limbic, frontoparietal, default-mode, and salience networks was the most compromised by AD compared with more sensory–motor networks. Moreover, the time-irreversibility properties associated with cognitive decline and atrophy outperformed and complemented classical neurocognitive markers of AD in predictive classification performance. Findings were generalized and replicated with an out-of-sample validation procedure. We provide novel multilevel evidence of reduced irreversibility in AD brain dynamics that has the potential to open new avenues for understating neurodegeneration in terms of the temporal asymmetry of brain dynamics.

## Introduction

*The irreversibility [of time] is the mechanism that brings order out of chaos*

(Prigogine and Stengers, 1984)

From physics to biology, the second law of thermodynamics states that closed systems evolve in the direction of entropy increases. A central consequence of the second law is the appearance of an asymmetry in the flow of temporal events that leads to the thermodynamic “arrow of time” (Eddington, 1928; Schrödinger, 1929). Such asymmetry distinguishes between reversible and irreversible/nonequilibrium (Seif et al., 2021). In the context of recent brain studies, large-scale self-organizing brain dynamics evolve away from thermodynamic equilibrium and remain in a healthy regime of multistability (Fingelkurts and Fingelkurts, 2004; Deco et al., 2017; Demirtaş et al., 2019; Luppi et al., 2019; Perl et al., 2021), where a positive correlation exists between neural dynamic complexity and the extent of temporal irreversibility, indicative of an association between healthy brain activity and nonequilibrium dynamics (Deco et al., 2022). Notably, this temporal signature reflects the complexity of the brain's functional organization and has been shown to relate to the level of consciousness (Perl et al., 2021; de la Fuente et al., 2022), cognitive performance (Deco et al., 2021; Lynn et al., 2021; Ibanez, 2022), and certain neuropsychiatric diseases (Zanin et al., 2019).

Across hundreds of studies, Alzheimer's disease (AD) has been characterized by an abnormal decrease in the complexity of brain activity, diminishing the entropy generation rate of neural dynamics (for review, see Sun et al., 2020). These imbalances in neural dynamics affect both cognition (Chand et al., 2018) and the intrinsic connectivity of resting-state networks (RSNs) across temporal levels [electroencephalography (EEG): Dottori et al., 2017] and spatial levels [functional magnetic resonance imaging (fMRI): Moguilner et al., 2021; see also both EEG and fMRI in the same study: Herzog et al., 2022)]. Therefore, the deviation from the expected level of entropy in the healthy brain may also involve substantial deviations from nonequilibrium, making brain dynamics in dementia less irreversible on different spatiotemporal scales. Despite this apparent connection, to our knowledge, no previous study has explored the arrow of time in the brain dynamics of AD patients.

Based on these considerations, we hypothesized that the level of irreversibility (i.e., the temporal asymmetry of brain dynamics and the variability of the global level of irreversibility over time), reflecting the nonstationarity nature of brain signals, would be compromised in AD patients. First, we anticipate that less irreversible brain dynamics would characterize AD across spatial (fMRI) and temporal (EEG) domains, from the global level (large-scale networks) to local regions and including different oscillatory frequency bands. Moreover, we propose that the more endogenous RSNs (i.e., default-mode, limbic, salience, and frontoparietal networks)—usually compromised in AD (Zhou and Seeley, 2014; Badhwar et al., 2017; Kucikova et al., 2021; Meng et al., 2022)—would show less irreversible dynamics than the more exogenous ones (somatosensory, motor, and visual networks) in AD patients. Second, we expect the level of irreversibility to be partially associated with classical neurocognitive markers of AD. We foresee an association of irreversibility with functional connectivity (FC) of RSNs and cognitive impairment. No specific associations would be observed between the level of irreversibility in AD and less transient measures such as brain atrophy. Finally, if less irreversible dynamics are a core signature of AD, the predictive classification power of multimodal irreversibility features (fMRI and EEG) would be similar to or greater than standard disease markers, including brain atrophy, functional brain connectivity, and cognitive deficits. Combining standard markers and irreversibility features would increase the classification power, suggesting that the latter measure provides additional information that the classic markers do not explain. We expect our measures to generalize to other unseen cohorts.

**Figure 1.**
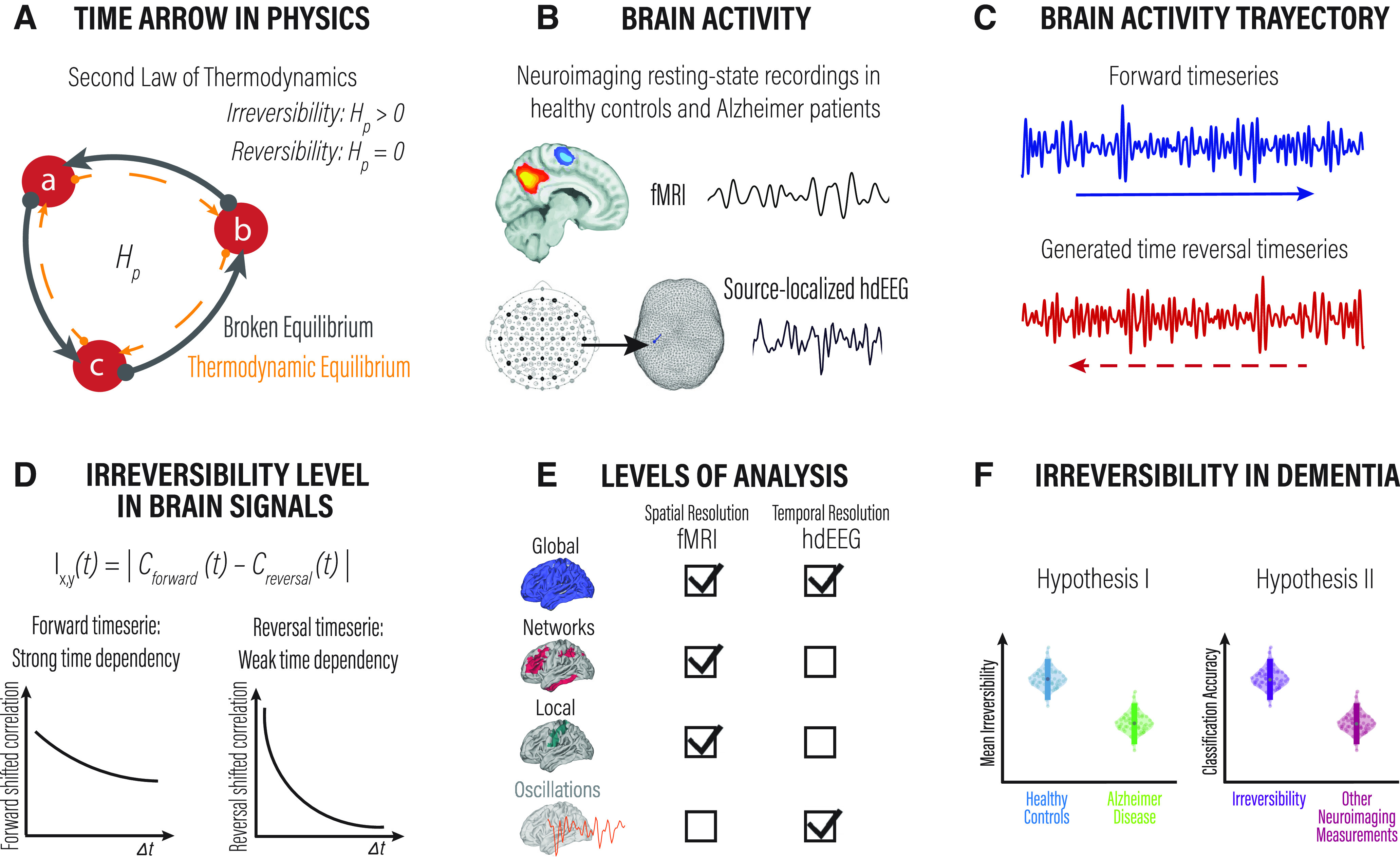
Overview of the framework to estimate the arrow of time in brain dynamics. We used a framework inspired by stochastic thermodynamics to capture the departure from equilibrium in large-scale brain dynamics as a potential distinctive signature of AD. ***A***, The second law of thermodynamics accounts for the energy transfer direction often referred to in the concept of “the arrow of time.” This physical law establishes that the entropy of an isolated system left to spontaneous evolution does not decrease, explaining the irreversibility of natural processes. The panel shows a three-state system and its transitions illustrated by circles and arrows. A system at thermodynamic equilibrium does not produce entropy; therefore, there are no net probability fluxes of transitions between states leading to reversible dynamics. ***B***, We used resting-state fMRI and source-inverted hdEEG data from the same pool of elderly healthy control subjects and Alzheimer's disease patients. Both datasets were parcellated using the AAL atlas. ***C***, From all brain regions in all participants, we extracted the forward brain activity time series and generated the corresponding backward versions. ***D***, The framework estimates the arrow of time (i.e., the level of irreversibility/nonequilibrium) using pairwise and multidimensional time-shifted correlation measurements. The correlation among time series captures the interaction between them. The bottom plots illustrate the interaction among the forward (left) and reversal (right) time series for a given time shift ( Δt). As can be seen, the correlation declines faster for signals with weak compared with a strong time dependency. ***E***, The level of irreversibility/nonequilibrium was estimated at different spatial scales, from the global coverage of the entire brain, the level of networks, and the local level of individual brain regions. ***F***, Finally, we used this framework to evaluate the irreversibility of neuroimaging data from patients with neurodegeneration. We hypothesized that the arrow of time would be affected by neurodegeneration and that this measure could equal or even surpass standard neuroimaging measures in the classification of neurodegenerative diseases.

To test these hypotheses, we adopted a theoretical nonequilibrium thermodynamics-inspired framework (Deco et al., 2021, 2022; Perl et al., 2021; de la Fuente et al., 2022) and implemented the associated methods (Fig. 1) to capture the temporal asymmetry of brain dynamics acquired using fMRI and EEG in patients with AD and elderly healthy control subjects (HCs). We assessed the temporal irreversibility by comparing forward and backward time series through time-shifted correlations applied to multimodal neuroimaging data, seeking to identify the brain networks predominantly affected by neurodegeneration and their anatomic and neuropsychological correlates.

## Materials and Methods

### Participants

We analyzed data from 107 male and female participants, 42 AD patients, and 65 matched elderly HCs, for which resting-state high-density EEG (hdEEG) and fMRI were acquired. *Post hoc* analyses revealed a calculated statistical power of 0.9898 (1 – β error probability) for the fMRI population sample, and 0.9979 for the hdEEG population sample. Recruitment was conducted in clinical centers in Chile (Geroscience Center for Brain Health and Metabolism, Memory and Neuropsychiatric Clinic, Universidad de Chile; Center for Social and Cognitive Neuroscience, Universidad Adolfo Ibáñez) and Argentina (Centro de Neurociencias Cognitivas, Universidad de San Andres) as part of an ongoing EEG/fMRI approach (Donnelly-Kehoe et al., 2019; Salamone et al., 2021; Birba et al., 2022; Legaz et al., 2022) led by BrainLat (Latin American Brain Health Institute; Duran-Aniotz et al., 2022). Consensus groups diagnosed participants with AD following National Institute of NINCDS (National Institute of Neurological and Communicative Disorders and Stroke)–ADRDA (Alzheimer's Disease and Related Disorders Association) clinical criteria (McKhann et al., 1984; 2011). Diagnoses were supported by an extensive battery of neurologic and neuropsychiatric assessments (Piguet et al., 2011; Baez et al., 2014; Melloni et al., 2016; Santamaría-García et al., 2017), conducted in line with the Multi-Partner Consortium to Expand Dementia Research in Latin America (ReDLat) protocol (Ibanez et al., 2021a, b; Maito et al., 2023). Participants with a history of neurologic disorders, primary language deficits, psychiatric disorders, or substance abuse were excluded. Table 1 reports demographic information for the fMRI, hdEEG, and matched samples, respectively. The relevant Institutional Review Boards approved the study, and all participants provided written informed consent before participation following the Helsinki declaration, National Institutes of Health guidelines, and local regulations.

**Table 1:** Demographics and cognitive performance

	HCs	AD patients	Statistics
fMRI sample	*N* = 65	*N* = 42	
Gender (F/M)	41/24	26/16	${\text{χ}}^{2}=0.0149;p=0.9026$
Age (mean ± SD)	69.92 ± 7.89	76.85 ± 7.46	F=20.51 ; p=1.56e−05
Education (mean years)	14.89	10.45	F=25.57;p=1.81e−06
MoCA (mean)	25.95	15.53	F=163.08;p=5.32e−21
hdEEG sample	*N* = 25	*N* = 28	
Gender (F/M)	15/10	17/11	${\text{χ}}^{2}=0.0028;p=0.9576$
Age (mean ± SD)	72.16 ± 6.70	76.53 ± 7.61	F=4.87;p=0.0317
Education (mean years)	13.6	9.89	F=7.70;p=0.0076
MoCA (mean)	25.15	16.36	F=38.67;p=5.07e−07
Matched sample	*N* = 22	*N* = 22	
Gender (F/M)	13/9	13/9	${\text{χ}}^{2}$ = 0; p= 1
Age (mean ± SD)	71.86 ± 6.94	76.18 ± 7.7	F=3,82;p=0,0575
Education (mean years)	13.13	10	F=5.22;p=0.0274
MoCA (mean)	25.35	16.36	F=51.29;p=1.72e−08

F, Female; M, male. Data presented for fMRI, hdEEG, and Matched samples. Categorical variables were analyzed using Pearson’s χ^2^ test, and continuous variables were assessed using ANOVA with an level of 0.05.

### Participants for out-of-sample validation

For out-of-sample validation, we used data from the Alzheimer’s Disease Neuroimaging Initiative (ADNI) database (https://adni.loni.usc.edu/). We analyzed resting-state fMRI data from 206 participants, 91 AD patients, and 115 elderly HCs. Both clinical groups were matched in age, gender, and years of education (Table 2).

**Table 2:** Demographics and cognitive performance for the ADNI cohort for out-of-sample validation procedures

Out-of-sample fMRI-ADNI	HCs (*N* = 115)	AD patients (*N* = 91)	Statistics
Gender (F/M)	59/56	38/53	${\text{χ}}^{2}=1.85;p=0.17$
Age (mean ± SD)	72.75 ± 8.51	73.62 ± 7.95	F=0.56;p=0.4544
Education (mean years)	15.75	15.41	F=1.06;p=0.3039
MoCA (mean)	24.24	14.23	F=238.07;p=1.25e−35

Categorical variables were analyzed using Pearson's χ^2^ test, and continuous variables were assessed using ANOVA with an α level of 0.05.

### Neuropsychological assessment

The overall cognitive state of the participants was examined using the Montreal Cognitive Assessment (MoCA; Nasreddine et al., 2005). The MoCA evaluates multiple cognitive domains, including attention, concentration, orientation, memory, language, executive functions, visuospatial skills, abstraction, and calculation.

### Neuroimaging data acquisition and preprocessing

#### hdEEG

Electrical brain signals were recorded using a Faraday cage with a 128-channel 24 bit resolution system (Active Two, Biosemi) located according to the radial electrode placement system and sampled at 1024 Hz while participants sat still and awake with their eyes closed. Two electrodes placed at the right and left mastoids served for offline rereference. Electrodes at periocular locations served to control for blinks and eye movements. Analog filters were applied at 0.03 and 100 Hz to the raw hdEEG data. Recordings were then bandpass filtered between 0.5 and 40 Hz using a zero-phase shift Butterworth filter, rereferenced to the average of all channels, and downsampled to 512 Hz. Malfunctioning electrodes were removed, and their values were estimated from neighboring electrodes using weighted spherical interpolation. Artifacts induced by eye movements were corrected using independent component analysis (Kim and Kim, 2012) and a visual inspection protocol (Schandry and Montoya, 1996; Dirlich et al., 1997; Pollatos and Schandry, 2004; Terhaar et al., 2012; García-Cordero et al., 2017; Yoris et al., 2017, 2018; Salamone et al., 2018). Recording and preprocessing of resting-state data followed current standards for multicentric connectivity studies in dementia harmonization regarding recording procedures, data acquisition Brain Imaging Data Structure (BIDS), preprocessing pipeline (denoising, artifact removal, spatial normalization, data normalization), and connectivity at the source space (Prado et al., 2022). All data and statistical analyses were performed using custom code written in MATLAB (MathWorks) following standard procedures (García-Cordero et al., 2017; Yoris et al., 2017, 2018; Mikulan et al., 2018; Salamone et al., 2018; Josefsson et al., 2019; Amoruso et al., 2022; Legaz et al., 2022).

#### EEG source space

We used the standardized Low-Resolution Electromagnetic Tomography (sLORETA) method (Pascual-Marqui et al., 1994) to estimate the neural sources of the hdEEG recordings. sLORETA computes the standardized current density at each of 2394 voxels located in the cortical gray matter and the hippocampus of a reference brain (MNI 305, Brain Imaging Center, Montreal Neurologic Institute) based on the linear, weighted sum of the scalp electric potentials. The electrodes layout was registered onto the scalp MNI152 coordinates, with landmarks for registering the electrodes located at the nasion, inion, the left preauricular point, and the right preauricular point. The locations of landmarks and recording electrodes were expressed in millimeters using the Cartesian coordinate system. To calculate the transformation matrix sLORETA (direct operator for the inverse solution problem), we used a regularization method with a signal-to-noise ratio of 1. The standardized current densities maps were obtained using a three-shell spherical head model registered to the Talairach space and parcellated using the Automated Anatomical Labeling (AAL; Tzourio-Mazoyer et al., 2002), removing subcortical regions. Time-varying current densities computed at each time point were averaged among voxels belonging to the same AAL region, such that a single (mean) time series was obtained for each cortical region (Prado et al., 2022).

#### fMRI recordings

Data were acquired at two different centers following Organization for Human Brain Mapping (OHBM) recommendations (Smith et al., 2013; Nichols et al., 2017; Poldrack et al., 2017) with the following parameters.

##### Center 1.

Imaging was performed on a 3 T system (Skyra, Siemens) with a standard head coil. T1-rapid gradient echo volumes, parallel to the plane connecting the anterior and posterior commissures, were acquired with the following parameters: repetition time (TR) = 1700 ms; echo time (TE) = 2000 ms; flip angle = 8°; 208 slices; matrix dimension = 224 × 224 × 208; voxel size = 1 × 1 × 1 mm. Functional ep2d_bold pulse sequences (parallel to the anterior–posterior commissures) covering the whole brain were acquired sequentially intercalating pair-ascending first with the following parameters fMRI parameters: TR = 2660 ms; TE = 30 ms; flip angle = 90°; 46 slices; matrix dimension = 76 × 76 × 46; voxel size in plane = 3 × 3 × 3 mm; slice thickness = 3 mm; sequence duration = 13.3 min; number of volumes = 300.

##### Center 2.

Imaging was performed on a 3-T Phillips system with a standard head coil. T1-rapid anatomic 3D gradient echo volumes were acquired parallel to the plane connecting the anterior and posterior commissures, with the following parameters: TR = 8300 ms; TE = 3800 ms; flip angle = 8°; 160 slices; matrix dimension = 224 × 224 × 160; voxel size = 1 × 1 × 1 mm. Functional spin echo volumes (parallel to the anterior–posterior commissures) covering the whole brain were sequentially and ascendingly acquired with the following parameters: TR = 2640 ms; TE = 30 ms; flip angle = 90°; 49 slices; matrix dimension = 80 × 80 × 49; voxel size in plane = 3 × 3 × 3 mm; slice thickness = 3 mm; sequence duration = 10 min; number of volumes = 220.

#### fMRI preprocessing

The data were preprocessed using the Data Processing Assistant for Resting-State fMRI (DPARSF version 2.3), an open-access toolbox with a standardized pipeline that uses Statistical Parametric Mapping software (SPM12) and the Resting-State fMRI Data Analysis Toolkit (REST version 1.7). The first five volumes of each subject’s resting-state recording were discarded to ensure that magnetization achieved a steady state. The preprocessing included slice-timing correction (using the middle slice of each volume as the reference scan) and realignment to the first scan of the session to correct head movement (implemented in SPM12; García-Cordero et al., 2016). Six motion parameters were regressed out: CSF and white matter (WM) signals to reduce potential effects of movement-related, physiological, and cardiorespiratory effects (REST version 1.7 toolboxes). Motion parameters were estimated during realignment, and CSF and WM masks were derived from the tissue segmentation of each subject’s T1 scan in native space with SPM12 (after coregistration of each subject’s structural image with the functional image). None of the participants showed head movements >3 mm and/or rotations >3°. Finally, images were normalized to the MNI space using the echoplanar imaging template from SPM12, smoothed using an 8 mm full-width-at-half-maximum (FWHM) isotropic Gaussian kernel, and bandpass filtered between 0.01 and 0.1 Hz to correct and remove low-frequency drifts from the MRI scanner. Data were parcellated with the AAL (AAL90; Tzourio-Mazoyer et al., 2002). There is substantial disagreement on global signal regression (GSR) in fMRI preprocessing. Given the potential bias that can be introduced in data analysis, we choose not to remove it. We based our decision on growing evidence supporting that GSR contains valuable information, including signals that correlate with electropshysiological sources and with vigilance fluctuations, among other signals of neurobiological relevance (Schölvinck et al., 2010; Wong et al., 2013; Chang et al., 2016; Wen and Liu, 2016; Liu et al., 2017; Turchi et al., 2018).

### Atrophy maps

MRI acquisition and preprocessing are reported following OHBM recommendations (Smith et al., 2013; Nichols et al., 2017; Poldrack et al., 2017). Whole-brain T1-rapid anatomic three-dimensional gradient echo volumes were acquired. For voxel-based morphometry (VBM) analysis, data were processed on the DARTEL Toolbox following validated procedures (Ashburner and Friston, 2000; García-Cordero et al., 2016; Sedeño et al., 2017) via SPM12 (https://www.fil.ion.ucl.ac.uk/spm/software/spm12/). T1-weighted images in native space were segmented using the default parameters of the SPM12 (bias regularization, 0.001; bias FWHM, 60 mm cutoff) into gray matter, white matter, and CSF. These three tissues were used to estimate the total intracranial volume (TIV). The DARTEL module was run using the gray matter-segmented and white matter-segmented images to create a template from the complete dataset (increasing the intersubject alignment accuracy; Ashburner, 2007). Next, the DARTEL Tool “Normalize to MNI Space” was used to register the last template from the previous step in MNI space. This transformation was applied to bring all the gray matter-segmented scans to standard space. Subsequently, all images were modulated to correct volume changes by Jacobian determinants and to avoid bias in the intensity of an area because of its expansion during warping. Finally, data were smoothed using a 10 mm FWHM isotropic Gaussian kernel to accommodate anatomic intersubject variability. The size of the kernel was selected based on previous recommendations (Ashburner and Friston, 2000; Burton et al., 2004). To analyze all images together, avoiding a scanner effect, the normalized and smoothed DARTEL images were transformed into *w-*score (Jack et al., 1997; La Joie et al., 2012; Ossenkoppele et al., 2015; Chung et al., 2017; van Loenhoud et al., 2017). *w-*Scores represent the degree to which the observed gray matter volume in each voxel is higher or lower than expected, based on an individual’s global composite score adjusted for specific covariates (age, TIV, and scanner type). *w-*Scores were calculated by dividing each participant’s observed and predicted gray matter volume (residuals) by their SD. The resulting *w-*score maps of each subject were used for further analyses.

### Method for determining levels of irreversibility/nonequilibrium

The level of irreversibility relies on the key idea of detecting the arrow of time through the degree of asymmetry obtained by comparing the lagged correlation between pairwise time series (i.e., the forward and the artificially generated reversed backward version). Figure 1 presents the general framework used to estimate the arrow of time and to provide a distinctive signature of health and disease in human brain recordings.

The detection of the level of irreversibility (i.e., the arrow of time) between two time series (*t*) and (*t*) requires creating the backward version of the time series. The reversed backward version of [*t* or (*t*)], that we call $x^{r}(t)$ [or $y^{r}(t)$], is obtained by flipping the time ordering [i.e., by ordering the time evolution of $x^{r}(t)$ (or $y^{r}(t)$)] as the inverted sequence determined the initial state and final states. The potential causal dependency between the time series (*t*) and (*t*) is measured through the time-shifted correlation (Deco et al., 2022). The extent to which the forward and reversed time series are distinguishable determines the reversibility/equilibrium level. Therefore, when the forward and reversed time series are not distinguishable, the system is reversible and in equilibrium, whereas when the level of distinguishability increases, the system becomes more irreversible and closer to nonequilibrium.

For each participant, we extracted forward BOLD time series from the AAL parcellation using sliding windows of 20 TRs, which were then shifted 1 TR forward (2.6 s, according to the acquisition parameter). We chose a time window of 20 TRs because it allows a good balance between sensitivity and specificity in detecting dynamic changes in correlations (Lindquist et al., 2014). However, we tested our approach with different TRs (30, 40, and 50 TRs).

The same strategy was applied to the source-localized broadband-filtered (0.5–40 Hz) EEG data but shifted 2 TRs forward (∼4 ms). EEG time series were also filtered in the five canonical frequency bands [i.e., δ, 0.5–4 Hz; theta, 4–8 Hz; alpha, 8–12 Hz; beta, 12–30 Hz; and gamma, 30–40 Hz), with the following time shift length: *Δt* = 15, 7, 5, 3, and 2, respectively. The time shift lengths were defined as a function of the decay of the signal autocorrelation. Then, we generated the backward version of each sliding window by reversing them in time. For the forward evolution, the time-shifted correlation is given by the following:

$$C_{\text{forward}}(\text{Δ}t)=<x\left(t\right),y(t+\text{Δ}t)>.$$

And for the reversed backward evolution, the time-shifted correlation is given by the following:

$$C_{\text{reversal}}(\text{Δ}t)=<x^{\left(r\right)}(t),y^{\left(r\right)}(t+\text{Δ}t)>.$$

The pairwise level of irreversibility (i.e., the degree of temporal asymmetry capturing the arrow of time) is given consequently by the absolute difference between these two time series in the forward and reversed backward evolution, at a given shift *Δt* = T, as follows:

$$I_{xy}\left(\text{T}\right)=\left|c_{\text{forward}}\left(T\right)-c_{\text{reversal}}\left(T\right)\right|$$

The level of irreversibility/nonequilibrium for the global/multidimensional case can be generalized by defining the forward and reversal matrices of time-shifted correlations. Let $x_{i}\left(t\right)$ indicate the forward version of a multidimensional time series reflecting the dynamic evolution of the variable describing the system. In this case, the subindex *i* denotes the different dimensions of the dynamic system (i.e., the brain regions). Let us denote with $x_{i}^{\left(r\right)}\left(t\right)$ the corresponding reversed backward version. Then, $x_{i}$ and $x_{j}$ refer to the forward time series in regions *i* and *j*, and $x_{i}^{\left(r\right)}\left(t\right)$ and $x_{j}^{\left(r\right)}\left(t\right)$ to the reversed backward time series, both time shifted. The forward and reversal matrices expressing the causal dependencies between the different variables for the forward and artificially generated reversed backward version of the multidimensional system are given by the following:

$$FS_{\text{forward,ij}}\left(\Delta t\right)=-\frac{1}{2}\text{log}(1-<x_{i}\left(t\right),x_{j}\left(t+\Delta t\right){>}^{2})$$

$$FS_{\text{reversal,ij}}\left(\Delta t\right)=-\frac{1}{2}\text{log}(1-<x_{i}^{\left(r\right)}\left(t\right),x_{j}^{\left(r\right)}\left(t+\Delta t\right){>}^{2})$$

The *FS* functional dependency matrices are expressed as the mutual information based on the respective time-shifted correlations. The level of irreversibility is given by the quadratic distance between the forward and reversal time-shifted matrices at a given shift Δt=T. In other words, the level of irreversibility in the multidimensional case is given by the following:

$$I=FS_{\text{forward}}(T)-FS_{\text{reversal}}{\left(T\right)}_{2}$$

Where the notation $Q_{2}$ is defined as the mean value of the absolute squares of the elements of the matrix *Q*. In other words, if we define a difference matrix $FS_{\text{diff}}$ in the following way:

$$FS_{\text{diff}}ij={\left(FS_{\text{forward}},ij\left(T\right)-FS_{\text{reversal,}}\text{ij}(T)\right)}^{2}$$

Then, the elements of the matrix $FS_{\text{diff}}$ are the square of the elements of the matrix $\left(FS_{\text{forward}}\left(T\right)-FS_{\text{reversal}}\left(T\right)\right),$ where for each pair, the level of irreversibility is measured by the squared difference. Thus, I is simply the mean value of the elements of $FS_{\text{diff}}$. We also computed the SD of the elements of the matrix $FS_{\text{diff}}$. The level of irreversibility for the local (nodal) case was estimated as the following:

$$I_{i}=\frac{1}{n}\underset{j}{\sum }{\left(FS_{\text{forward}}ij(T)-FS_{\text{reversal}}ij(T)\right)}^{2}\text{.}$$

### fMRI functional connectivity

The functional connectivity are matrices of Pearson correlation coefficients across the entire duration of the BOLD time series of the 90 AAL brain regions (Friston, 1994).

### EEG functional connectivity

Functional interaction between brain regions within a set frequency band was estimated using EEG coherence analysis. Coherence estimates relative amplitude and phase consistency among all pairs of data channels (Srinivasan et al., 2007).

### Statistical analyses

Categorical variables (i.e., gender) were analyzed using Pearson's χ^2^ test, and continuous variables (i.e., age, years of education, and cognitive performance) were assessed using ANOVA. Demographic differences were controlled by performing a multivariate linear regression analysis on irreversibility at the global level. Differences between conditions in irreversibility were statistically assessed across subjects and across brain regions using the Wilcoxon rank-sum method. Additionally, we used the false discovery rate (FDR) at the 0.05 significance level to correct multiple comparisons (Hochberg and Benjamini, 1990). Changes in irreversibility at the network and local level were also assessed using Cohen’s *d* effect size, which measures the effect size in terms of the difference between the means of two populations (μ1, μ2) and the pooled SDs (*s*) as follows: $d=\frac{\text{μ}1-\text{μ}2}{s}$. Following a standard criterion to relate Cohen’s *d* values to the effect size, *d *<* *0.2 is considered a very low effect size, while *d* > 0.8 represents a very high effect size (Sawilowsky, 2009). Correlations between the irreversibility measurements with atrophy, cognitive performance, and functional connectivity were tested with Pearson’s *R* test and the associated *p*-values (with Benjamini–Hochberg correction). Subjects with irreversibility values that were more than three scaled median absolute deviations from the median were excluded from the analysis.

### Random forest classifier

The *fitcensemble* MATLAB function was used with default parameters to train a random forest classifier with the purpose of distinguishing between the groups of participants. To perform cross-validation, the dataset was randomly partitioned with stratification of *k *=* *5. Then, the model was fitted using four of these groups (training/test = 80/20), and its performance was tested using the group that remained. This procedure was repeated 50 times to obtain distributions and average measures of performance. The features used were the following: EEG irreversibility (NR); fMRI NR, fMRI, and hdEEG NR (combined); mean FC, atrophy, cognitive performance (MoCA results); all brain functional measurements (EEG/fMRI NR, FC); and multimodal measurements (EEG/fMRI NR, FC, atrophy, and cognitive performance). Missing data were estimated using the weighted K-nearest neighbors (KNNimpute; Troyanskaya et al., 2001), with *k* = 5. KNNimpute is a weighting procedure that exploits the correlation between a missing value and the available data, quantified using the support vector regression method. Classification performance was estimated using the receiver operating characteristic (ROC) area under the curve (AUC), precision, sensitivity, specificity, accuracy, and *F*-score metrics (Fawcett, 2006).

## Results

### Alzheimer's disease is associated with significant changes in the level of irreversibility/nonequilibrium in multimodal spatiotemporal dynamics

**Figure 2.**
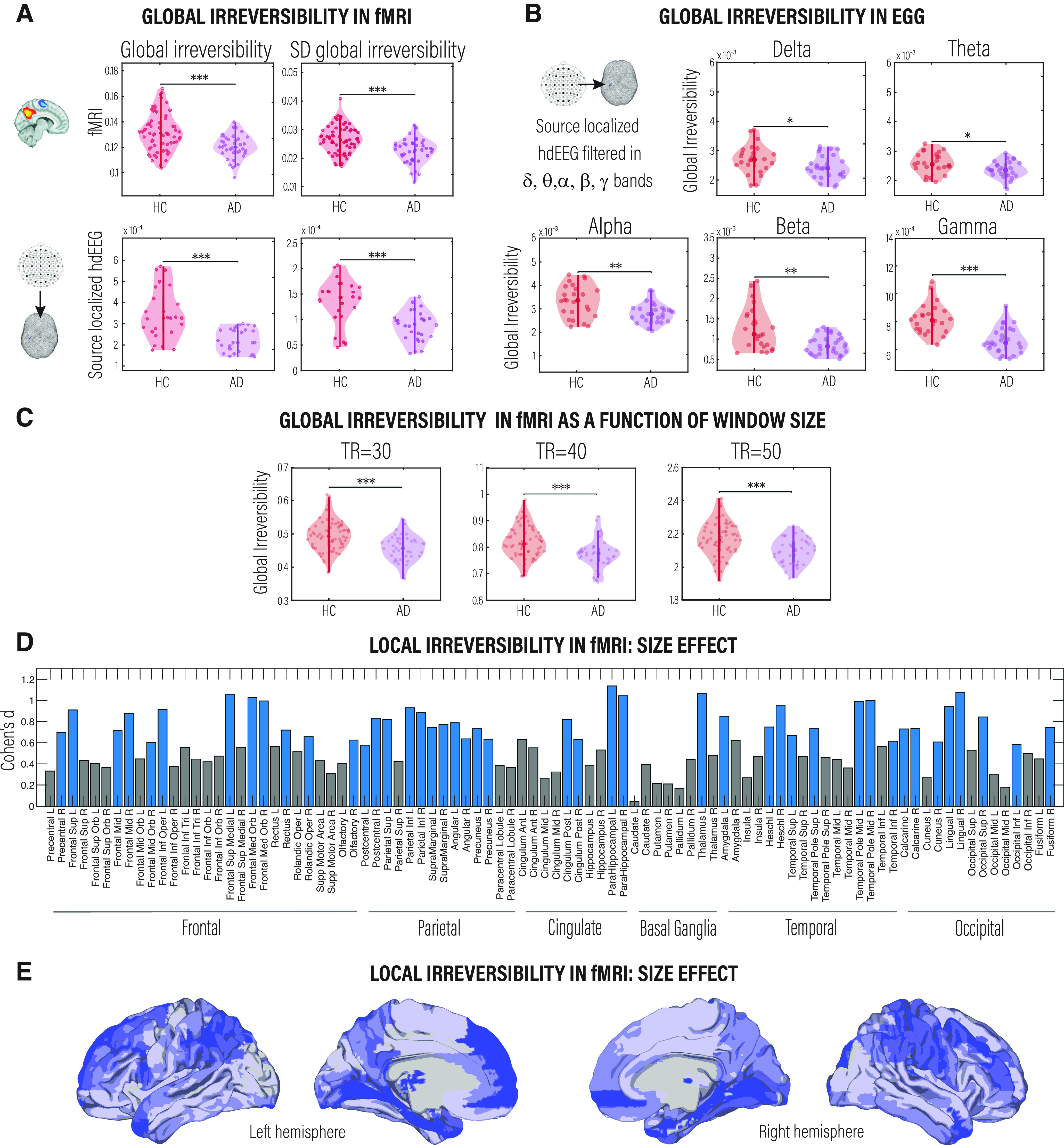
Irreversibility/nonequilibrium in large-scale brain signals as a signature of neurodegenerative diseases. ***A***, Shows the global level of irreversibility and the variability of the global level of irreversibility for HCs compared with AD patients estimated in fMRI (top left and top right, respectively) and source-localized hdEEG data, broadband filtered (0.5–40 Hz; bottom left and bottom right, respectively). ***B***, Comparison of the proximity to nonequilibrium brain dynamics between conditions for the source-localized hdEEG dataset filtered in frequency bands (delta, theta, alpha, beta, and gamma). All *p*-values were estimated using the Wilcoxon rank-sum test and FDR corrected for multiple comparisons with an α level of 0.05; *p < 0.05, **p < 0.01, and ***p < 0.001. ***C***, Global irreversibility as a function of the window size (time length) in which the forward BOLD time series were extracted. ***D***, Rendering of the effect size (estimated by Cohen’s *d*) of the level of the irreversibility of brain activity at each node defined by the AAL atlas. Blue bars indicate brain regions showing significant differences (*p* < 0.01, Wilcoxon rank-sum test, FDR corrected) between HCs and AD patients. ***E***, As in ***C***, the brain plots show the effect size (estimated by Cohen’s *d*) of the level of the irreversibility of brain activity at each node.

First, we explored whether the arrow of time estimated from fMRI was compromised in AD at a global level and whether the level of irreversibility/nonequilibrium could distinguish between HCs and AD patients. We found that the irreversibility in neural dynamics differentiated HCs from AD patients (Fig. 2*A*, left panels; *p* < 0.001, Wilcoxon rank-sum test, FDR corrected). In addition, we found that the variability of the global level of irreversibility over time was also distinguished between conditions (Fig. 2*A*, right panels; *p* < 0.001, Wilcoxon rank-sum test, FDR corrected). We further complemented these results using the source-localized hdEEG data filtered in the five canonical frequency bands (all *p* < 0.05, Wilcoxon rank-sum test, FDR corrected: delta, 0.5–4 Hz; theta, 4–8 Hz; alpha, 8–12 Hz; beta, 12–30 Hz; and gamma, 30–40 Hz), with a time shift length according to the frequency band (TR = 15, 7, 5, 3, and 2, respectively). We observed significant differences between groups at all frequency bands (Fig. 2*B*). In addition, we calculated the cross-modal correlation coefficient of the global irreversibility level and found no relationship (HC + AD: *R* = 0.04, *p* < 0.08.033; HC: *R* = 0.04, *p* < 0.8651; AD: *R* = 0.04, *p* < 0.8637).

To rule out that our results depend on the length of the time window of BOLD time series, we estimated the level of irreversibility using three different TRs (30, 40, and 50) and replicated previous results (Fig. 2*C*). We also ruled out the possible influence of age and education (Table 1) on the results; we applied a multivariate linear regression analysis on irreversibility at the global level. This procedure replicated previous global results for the fMRI and EEG samples (*p* < 0.001, Wilcoxon rank-sum test; fMRI: *r *=* *0.9877, *p *<* *0.001; EEG: *r *=* *0.9993, *p *<* *0.001), suggesting that although temporal asymmetry in brain dynamics might be related to demographic variables, such as age and education, the level of irreversibility reflected changes mainly based on brain dynamics.

To capture the irreversibility/nonequilibrium changes at the node level, we applied the framework at each BOLD time series extracted from the AAL parcellation. We observed that the difference in irreversibility was significantly higher in bilateral frontal and temporoparietal regions in HCs than in AD patients. Figure 2*D* shows the magnitude of this difference, and Figure 2*E* highlights the areas presenting significant differences between conditions (*p* < 0.01, Wilcoxon rank-sum test, FDR corrected). Finally, to explore whether the size of the ROIs explains their level of irreversibility, we correlated the irreversibility at the local level with the size of each ROI (estimated as the number of voxels) and found that there is no relationship between them for any of the two conditions or techniques (HCs: fMRI, *R* = −0.00096047, *p* < 0.41202; EEG, *R* = 0.015287, *p* < 0.3658; AD patients: fMRI, *R* = −0.0031627, *p* < 0.50 261; EEG, *R* = 0.010345, *p* < 0.35 476).

### Selective compromise of irreversibility across critical networks involved in neurodegeneration

Beyond the global and local analyses, we investigated the differences in the level of irreversibility associated with seven RSNs. To do so, we applied the same framework for the global level in the BOLD time series but now restricted it to brain regions in each of the seven RSNs.

**Figure 3.**
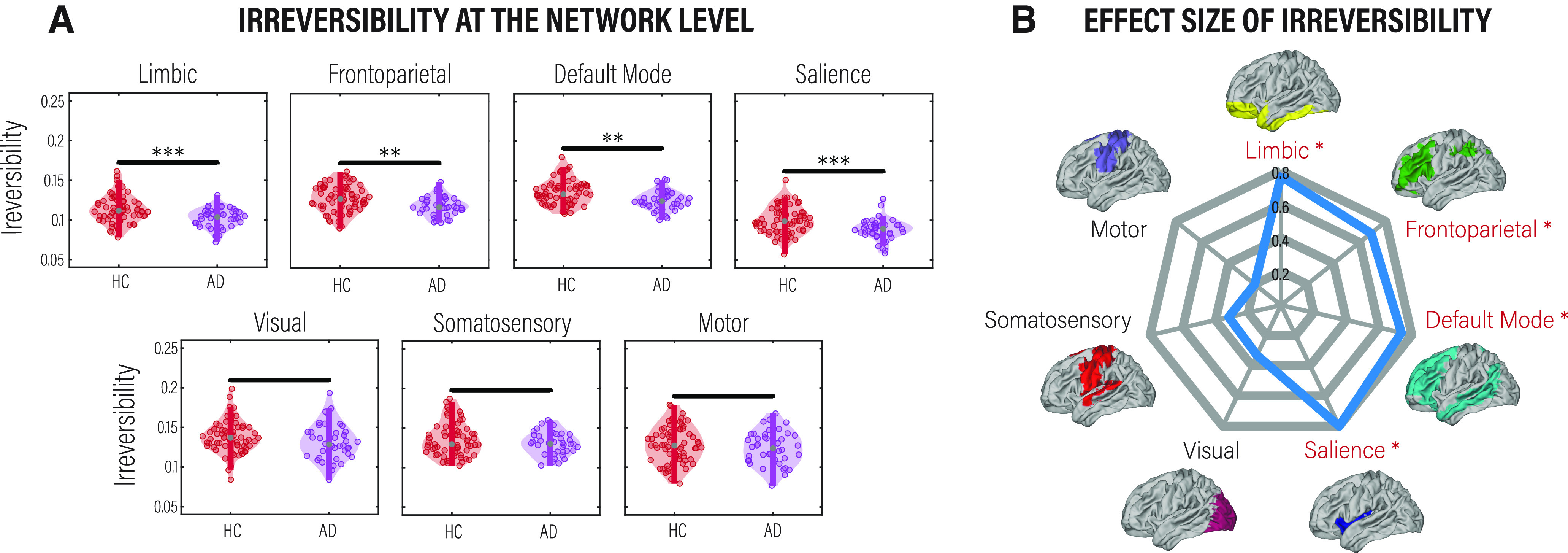
Level of irreversibility/nonequilibrium in fMRI resting-state networks. We followed the same procedure used in the global analysis but now in each subset of regions belonging to each of the seven networks. ***A***, Comparison of the level of irreversibility for each network between HCs and AD patients. *p*-values were estimated using the Wilcoxon rank-sum test and FDR corrected for multiple comparisons with an α level of 0.05. **p < 0.01 and ***p < 0.001. ***B***, Rendering of the effect size assessed by Cohen’s *d*. Names in red with an asterisk indicate networks showing significant differences between conditions.

The RSNs we considered involve some that are typically impaired in AD (i.e., limbic, frontoparietal, DMN, and salience) as well as other that act as control (i.e., somatosensory, visual, motor). We found decreases in the irreversibility level in AD patients compared with HCs only in networks known to be affected by AD: limbic, frontoparietal, DMN, and salience networks, which are also more endogenous networks (less driven by the external environment). Conversely, no significant differences were found in primary sensorimotor networks (visual, somatosensory, and motor networks). This result suggests that the impairment in sustaining the nonequilibrium is evident in the selective physiopathology of ADs, indexing different signatures in the balance of intrinsic and extrinsic brain dynamics. Figure 3*A* shows the differential responses between HCs and AD patients for the RSN by presenting boxplots (*p* < 0.05, Wilcoxon rank-sum test, FDR corrected). Figure 3*B* presents a combined radar plot illustrating the effect size of the different levels of irreversibility for each resting-state network.

### Associations of irreversibility with neurocognitive markers of Alzheimer’s disease

**Figure 4.**
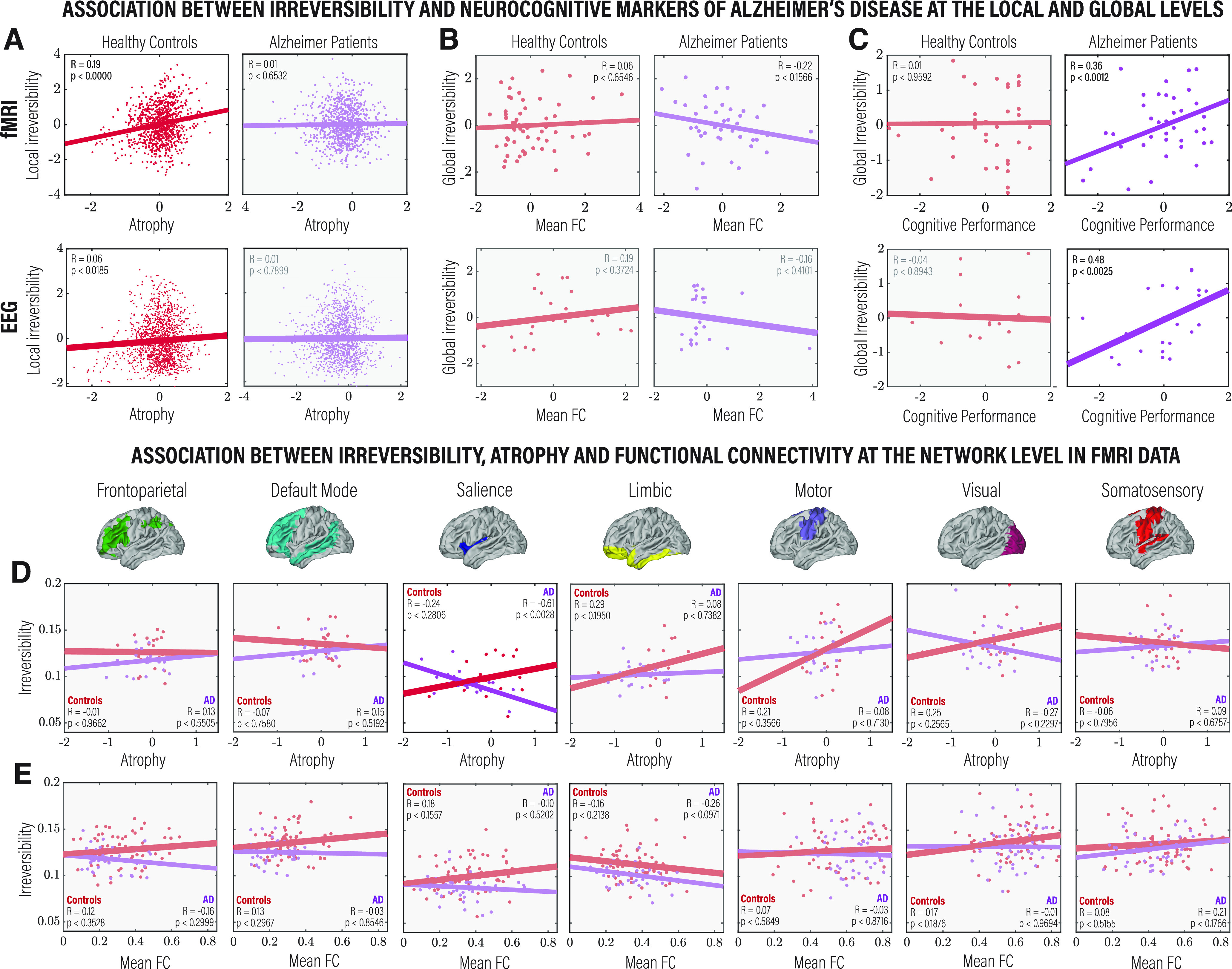
Comparison between levels of irreversibility estimated from fMRI and source-localized hdEEG data and standard structural, functional, and behavioral measurements, as well as with the local, global, and network levels. ***A***, Correlation between irreversibility and gray matter volume (w-scores) at the node level in brain areas showing a significant difference in irreversibility between HCs and AD patients, for HCs (left) and AD patients (right), and for fMRI (top) and hdEEG (bottom). ***B***, Association between global irreversibility and mean FC. ***C***, Correlation between global irreversibility and cognitive performance estimated using the MoCA. ***D***, Correlation between irreversibility and gray matter volume (w-scores) for each of the seven RSNs between HCs and AD patients. ***E***, Association between global irreversibility and mean functional connectivity. All metrics are reported in *z* scores. Pearson’s *R* and the associated *p-*values are reported for each comparison and were FDR corrected for multiple comparisons with an α level of 0.05.

Since AD is usually associated with specific patterns of atrophy, cognitive deficits, and alterations in functional connectivity, we explored whether the extent of irreversibility in the dynamics—estimated from both fMRI and EEG—was related to these variables. First, we related the irreversibility at the node level to atrophy, estimated as gray matter volume. We found a significant correlation between irreversibility and atrophy for HCs (fMRI: *R* = 0.19, *p* < 0.001; EEG: *R* = 0.06, *p* < 0.0185; FDR corrected) but not for AD patients (fMRI: *R* = 0.01, *p* < 0.6532; EEG: *R* = 0.01, *p* < 0.7899; FDR corrected), suggesting that the emergence of the arrow of time in brain dynamics is not primarily linked to atrophy in AD (Fig. 4*A*). Then, we explored the association with the mean functional connectivity, for which no significant results were found for HCs (fMRI: *R* = 0.06, *p* < 0.6546; EEG: *R* = 0.19, *p* < 0.3724; FDR corrected) and for AD patients (fMRI: *R* = −0.22, *p* < 0.1566; EEG: *R* = −0.16, *p* < 0.4101; FDR corrected; Fig. 4*B*). We then investigated the correlation between global irreversibility and cognitive performance using the MoCA. The results showed a significant correlation between the two measures in AD patients (fMRI: *R* = 0.36, *p* < 0.0012; EEG: *R* = 0.19, *p* < 0.3724; FDR corrected) but not in HCs (fMRI: *R* = 0.01, *p* < 0.9592; EEG: *R* = −0.16, *p* < 0.4101; FDR corrected; Fig. 4*C*). Finally, we restricted the association of irreversibility, atrophy, and FC to each of the seven RSNs and found solely a correlation between the irreversibility and atrophy in brain regions belonging to the salience network (*R* = 0.24, *p* < 0.2806, FDR corrected) in AD patients (Fig. 4*D*,*E*). No significant associations were found between the other RSNs and atrophy or functional connectivity levels.

### Comparing the selective and combined classification power of irreversibility dynamics with standard neurocognitive markers

**Figure 5.**
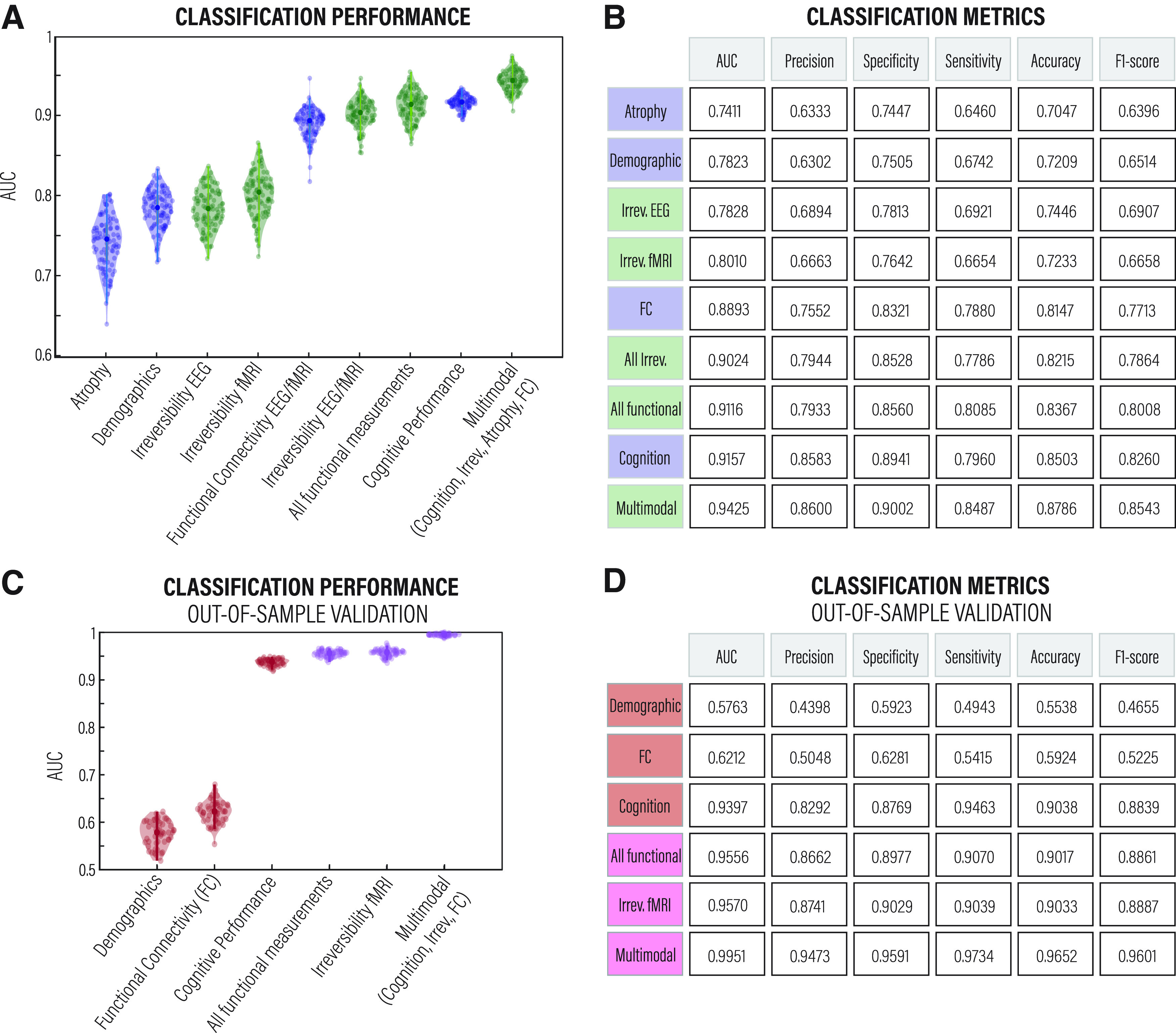
Classification accuracy. ***A***, ROC AUC obtained from a random forest classifier trained using eight different independent features and an additional multimodal feature comprising cognitive performance, irreversibility estimated from EEG and fMRI data, atrophy, and functional connectivity, also estimated from EEG and fMRI data. ***B***, The performance of the classifier was assessed using the AUC, precision, specificity, sensitivity, accuracy, and *F*1-score, obtaining one average value for each feature. When incorporating the irreversibility values, the classification accuracy score was >90%, surpassing the cognitive performance (assessed with MoCA). These tests were conducted using a normal train/test split and without much parameter tuning. ***C***, ***D***, ROC AUC and the classification metrics, respectively, from an independent cohort for results validation. Green and pink show irreversibility features or multimodal features that comprise irreversibility. Blue and red show other neurocognitive features of AD.

Given the capacity of the irreversibility measure to distinguish between AD and HCs, we focused on its predictive power for classification accuracy. We tested the predictive power of the irreversibility measure in each modality against and in conjunction with other structural, functional, behavioral, and demographic measurements. The results revealed that the reversibility of the arrow of time estimated from fMRI and source-inverted hdEEG time series outperformed hdEEG/fMRI FC and atrophy measurements (Fig. 5*A*). Moreover, the highest classification was obtained by combining multimodal features, which suggests that irreversibility is a strong predictor of AD and brings information not covered by classical neurocognitive measures. Figure 5*B* presents the classification performance with multiple metrics.

### Generalization and replication with an out-of-sample validation

To test the generalization of our approach and rule out potential classification sample size biases in the dataset, we tested the arrow of time and the predictive power of the irreversibility measure on an independent dataset (i.e., the publicly available ADNI cohort). As shown in Figure 5, *C* and *D*, we replicated previous findings, further strengthening the results.

## Discussion

We used a thermodynamics-inspired framework to gauge the impact of AD on the temporal asymmetry of empirically extracted brain signals (i.e., the level of irreversibility/nonequilibrium in large-scale dynamics estimated from fMRI and hdEEG). The results showed that resting brain activity in AD is associated with a reduction in irreversibility at the global level of the entire brain, local level of cortical regions defined in the AAL parcellation, network level of the prototypical RSNs, and at different oscillatory regimes. Complementarily, the decrease in irreversibility was associated with cognitive decline in AD patients and atrophy in HCs. In addition, we observed that the irreversibility property of brain dynamics outperformed classical neurocognitive measures in predictive classification performance. In addition, multimodal features comprising irreversibility, FC, atrophy, and cognitive performance reached the highest classification performance, surpassing 90% accuracy. On the one hand, our findings offer novel multilevel evidence of reduced irreversibility in AD brain dynamics, reinforcing the close pathophysiological link between brain dynamics and the clinical presentation of AD. On the other, our findings have the potential to open new avenues for understating neurodegeneration in terms of entropic asymmetry of brain dynamics.

These results allow for bridging the gap between large-scale dynamics and AD in threefold ways. First, we show that AD drives brain dynamics toward a different thermodynamic regime (i.e., a state of equilibrium), making them less irreversible in time as an indicator of brain disease. In classical thermodynamics, irreversibility is associated with the production of entropy. A drastic decrease in the entropy of spontaneous brain activity, as in the case of AD (for review of multiple studies, see Sun et al., 2020; for review of conflicting evidence, see Xue and Guo, 2018), would render brain dynamics pathologically subcritical, reducing the net fluxes between the underlying states and thus hampering the integrity of the level of irreversibility—probably because of the large amount of energy required. Therefore, it seems unlikely that a system can sustain the dynamic nature of healthy cognitive functions without substantial deviations from thermodynamic equilibrium on some spatial and temporal scales. Consistent with the entropy production interpretation, brain activity requires a certain level of entropy to maintain its functional flexibility; then, its reduction would lead to impairments in cognitive functions and adaptive behavior (Tagliazucchi et al., 2012; Grieder et al., 2018; Saxe et al., 2018; Wang, 2020). Second, to our knowledge, this is the first report of irreversibility as a multimodal feature of neurodegeneration. We show that the extent to which brain dynamics deviate from the state of equilibrium is a powerful marker of AD. This feature proves more powerful than, and complementary to, more classical neurocognitive measures in distinguishing AD. And third, particularly noteworthy, our neuroimaging research provides convergent evidence supporting pathologic irreversibility in AD across different recording techniques, spatial levels of analysis, and oscillatory regimes.

Irreversibility clearly distinguished AD patients from HCs, and the metric is robust across all spatiotemporal scales. Specifically, we found lower levels of irreversibility and variability of the irreversibility in the BOLD signal at the global level in AD patients than in HCs. We corroborated the results in source-localized hdEEG data-filtered broadband and in the five canonical frequency bands (delta, theta, alpha, beta, and gamma). We showed that the irreversibility differences between HCs and AD patients remain across a range of window sizes. Similarly, we found lower levels of irreversibility at the local level of individual brain regions in the distributed frontal and temporoparietal areas. This fact indicates that the degree of temporal asymmetry is inherent to neural dynamics regardless of the data modality and the temporal and spatial scales of the analysis. Also, the fact that fMRI and EEG provide the same results points out the robustness of the method. However, despite consistent results across modalities, these results were not correlated. This could be because EEG and fMRI measure signals from different origins and timescales, and they show different but complementary proxies of brain activity that do not necessarily appear correlated—and even less so when they have not been recorded simultaneously (Warbrick, 2022). This result fuels the growing need in current neuroimaging research to understand the functional links between quantitative measures derived from EEG and the BOLD signal assessed with fMRI.

We further investigated the irreversibility at the network level using the same framework applied to the global level but restricted to brain regions in each of the seven RSNs estimated using fMRI. We found that the more endogenous networks (i.e., limbic, frontoparietal, default mode, and salience) showed the lowest levels of irreversibility in AD. These findings align with previous neuroimaging studies that support AD as a disconnection syndrome in which these networks operate in significant isolation (i.e., at greater connectivity distances from the rest of the brain; Brier et al., 2014; Costumero et al., 2020). The results also suggest that in AD, the high-order processing performed by the more endogenous RSNs is less dependent on the interaction between the system and its environment, reducing top-down processing. From a functional perspective, this could translate into deficiencies that may play a role in cognitive decline because of, for example, poor information processing guided by experience and expectations or lack of allocation of attention to relevant features.

Because of its excellent temporal resolution, EEG has long been the hallmark tool for investigating the rapid dynamics of brain electrical activity in healthy and diseased human brains, enabling valuable scientific contributions to various research fields. However, among the methodological disadvantages of this technique is the limited spatial resolution and, thus, the difficulty of obtaining precise source locations. For this reason, in this work, we refrained from reporting EEG brain hubs and networks similar to those detected by fMRI and instead preferred to exploit and maximize their temporal strength by analyzing the five canonical frequency bands. Future studies may overcome this limitation by using 256 hdEEG electrodes as the spatial resolution of source reconstruction procedures improves localization accuracy with more dense-array sampling (Song et al., 2015).

When evaluating the association of the level of irreversibility, estimated from both fMRI and EEG data, with classical neurocognitive markers of AD, we found a significant correlation between node-level irreversibility and atrophy values in HCs, but not in AD patients; and between global-level irreversibility and cognitive performance in AD patients, but not in HCs. It is noteworthy that the results were consistent across recording techniques. In healthy brains, structural measures have an expected association with irreversibility/nonequilibrium dynamics, but these are abolished in AD. Likewise, cognitive performance—potentially related to multiple processes in healthy control subjects—is associated explicitly with core cognitive impairments in AD, suggesting a specific role of disrupted irreversibility in neurodegeneration and cognitive deficits. At the network level, an association between irreversibility and atrophy was found only in brain regions of the salience network in AD. The salience network identifies salient stimuli and switches between the frontoparietal network and the default mode network, both of which are impaired in AD (Agosta et al., 2012; Zhao et al., 2018, 2019). Alterations in functional connectivity in the salience network have also been related to AD (Balthazar et al., 2014; Lee et al., 2020). Moreover, these results suggest that the structural mechanisms related to dynamic switching between transient states are associated with less irreversible dynamics in AD. No associations were found between irreversibility and FC, suggesting that irreversible dynamics have different/unrelated properties to standard resting-state connectivity measurements—as supported by the higher classification performance of the former. Although somewhat unexpected, this result also supports previous studies focused on linear correlations of resting-state data that agree that this measure may not provide a complete description of the temporal properties of the brain signal, as it systematically underestimates the strength of the dependence structure (LaConte et al., 2004; Hlinka et al., 2011).

Using a recently developed multifeature approach (Moguilner et al., 2022) and after exploring the arrow of time in the empirical data, we focused on the predictive power for classification accuracy aiming to compare the results of standard measurements used in dementia research with the irreversibility metrics on the same population and two independent samples, as a way to further assess the generalizability of our findings via the process of cross-validation. Our results were bolstered by the high discriminative performance obtained using multimodal irreversibility features (fMRI and EEG) in contrast to standard neurocognitive markers of AD, including brain atrophy (Moradi et al., 2015; Lebedeva et al., 2017), functional connectivity (Chen et al., 2011; Challis et al., 2015), and cognitive deficits (Ewers et al., 2012; Binaco et al., 2020). Results show that irreversibility carries information not covered by classical neurocognitive measures, suggesting that it is a critical property of AD. Moreover, the combination of multimodal measurements (FC, cognition, atrophy, irreversibility) further increased the classification performance, suggesting that the irreversibility measure provides differential information not explained by classical AD markers.

Regarding formulation of the irreversibility measure, our analysis is based only on the effect size of the correlations (*R*^2^ coefficient) regardless of their statistical significance. Introducing a threshold based on a statistical test could be problematic, given the large number of tests applied for each temporal window and thus the need to perform a strict correction for multiple comparisons, leading to potentially very sparse matrices. Since our analysis weights connections according to the correlation of the corresponding BOLD time series, correlations that are not significant will have small values that will fluctuate around zero, overall making a small amount to the temporal irreversibility estimate relative to the stronger connections. We believe there are no reasons to suppose that the potential inclusion of some spurious correlations will selectively affect one of the groups more than the others (i.e., that they will confound the results concerning differences between groups).

While similar to or larger than other studies, we acknowledge that our small sample size is a potential methodological limitation that might affect the reporting of our results. Still, it was minimized by the large effect sizes observed, and the rigorous control of clinical variables, diagnostic procedures, and assessments (Piguet et al., 2011; Rascovsky et al., 2011; Gil et al., 2015; Melloni et al., 2016). Although small sample sizes are common, they can be particularly problematic for machine learning, as overall accuracy might be higher in studies with smaller sample sizes (Arbabshirani et al., 2017; Varoquaux, 2018). Since the features of our study were derived from the same dataset, which could lead to information leakage in machine learning, we conducted an out-of-sample validation analysis using fMRI data from the ADNI cohort. We successfully replicated the irreversibility findings and classification accuracy using this unseen independent cohort. The combined results support the potential generalization of our approach. The combined results support the potential generalization capability of our approach. Still, given that we are amid a replicability crisis, in which many scientific findings are not replicated in new datasets, one must proceed cautiously. Generalizability goes hand in hand with replicability, and issues at all stages could emerge (i.e., data collection, preprocessing pipeline, and data analysis).

In addition, as a result of the sample size limitation, future studies should involve the application of this framework in a greater sample size and ideally consider different stages of the disease to tackle the evolution of irreversibility in brain dynamics. Indeed, our findings have the effects of the influential covariates of demographics. To rule out that the reduction in irreversibility was not because of them, we performed a multivariate linear regression analysis on irreversibility at the global level. The fact that we got the same results confirms the findings. In addition, the machine learning classification based on demography presented smaller effects compared with all other cognitive, structural, functional, and irreversibility markers. In addition, future studies should further explore the association of this synergistic approach with cognition (Ibanez, 2022) and with combined forms of brain structural characterization, different functional connectivity measurements, and other biomarkers, such as metabolic changes acquired with PET or additional emerging plasma and CSF biomarkers (Migeot et al., 2022). Finally, it would be of particular importance to contrast these results with those of other neurodegenerative diseases to assess whether we are tackling a specific AD signature or a general property of neurodegeneration.

The present work provides a framework derived from the perspective of thermodynamic equilibrium for assessing irreversibility in time series at different spatiotemporal scales. We showed that AD is associated with less irreversible brain dynamics in spatial and temporal domains, spanning the global-level, large-scale networks and local regions, and multiple oscillatory regimes. This signature correlates with prototypical neurocognitive markers of AD, such as cognitive decline, and it provides higher classification accuracy than classical neurocognitive markers of AD, especially when combined with other features. Overall, the results suggest that the irreversibility of the time series reflects a genuine hallmark of inefficient brain dynamics in AD and points to the link between selective and relentlessly progressive pathologic changes and entropic brain dynamics in AD.
